# Pre-B acute lymphoblastic leukemia expresses cell surface nucleolin as a 9*-O-*acetylated sialoglycoprotein

**DOI:** 10.1038/s41598-018-33873-2

**Published:** 2018-11-21

**Authors:** Eun Ji Joo, Brian R Wasik, Colin Parrish, Helicia Paz, Martina Mϋhlenhoff, Hisham Abdel-Azim, John Groffen, Nora Heisterkamp

**Affiliations:** 1 Department of Systems Biology, Beckman Research Institute, City of Hope, Monrovia, CA USA; 2 Department of Microbiology and Immunology, Baker Institute for Animal Health and Feline Health Center, Cornell University, Ithaca, NY USA; 3 Section of Molecular Carcinogenesis, The Saban Research Institute of Children’s Hospital Los Angeles, Los Angeles, CA USA; 4 Institute of Clinical Biochemistry, Hannover Medical School, Hannover, Germany; 5 Division of Hematology/Oncology and Bone Marrow Transplant, Children’s Hospital Los Angeles, Los Angeles, CA USA; 6 Departments of Pediatrics and Pathology, Keck School of Medicine, University of Southern California, Los Angeles, CA USA; 7 Present Address: University of California, Los Angeles, CA 90095 USA

## Abstract

Precursor B acute lymphoblastic leukemias (pre-B ALLs) abnormally express a specific glycan structure, 9-*O-*acetylated sialic acid (9*-O-*Ac-Sia), on their cell surface, but glycoproteins that carry this modification have not been identified. Using three different lectins that specifically recognize this structure, we establish that nucleolin (NCL), a protein implicated in cancer, contains 9-*O*-Ac-Sia. Surprisingly, antibodies against the glycolipid 9*-O-*Ac-Sia GD3 also detected 9*-O-*Ac-Sia NCL. NCL is present on the surface of pre-B ALL cells as a sialoglycoprotein that is partly 9-*O*-acetylated and conversely, 9-*O*-Ac-Sia-containing structures other than NCL are present on these cells as well. Interestingly, NCL and the 9*-O-*Ac-Sia signal had less co-localization on normal pre-B cells. We also investigated regulation of NCL on the cell surface and found that sialidase treatment increased the percentage of cells positive for cell surface NCL, suggesting that sialylation of NCL promotes internalization. Treatment of pre-B ALL cells with the chemotherapy drug vincristine also increased the percentage of cells with surface NCL and correlated with increased 9*-O-*Ac-Sia expression. All tested leukemia cells including primary samples expressed NCL, suggesting it as a possible therapeutic target. We confirmed this by showing inhibition of cell proliferation in some pre-B ALLs by exposure to a NCL-specific aptamer AS1411.

## Introduction

Precursor B-cell acute lymphoblastic leukemia (pre-B ALL) is the most prevalent malignancy of childhood^1^. Cell surface structures that selectively identify leukemia cells could be used for diagnosis or perhaps even treatment, and therefore previous studies reporting increased expression of a very specific glycan structure on pre-B ALL cells, consisting of sialic acid modified by 9-*O*-acetylation, are of significant interest in this context^2,3^.

Sialic acids (Sias) belong to a family of 9-carbon carboxylated monosaccharides that most frequently are found as the terminal residues of glycoconjugates, which include glycoproteins and glycolipids. Sias are often highly expressed in cancer^4,5^, and, moreover, changes in sialylation promote cancer progression^6^. Sialic acids can carry further modifications, of which *O*-acetylation is the most common, and preferentially occurs at the hydroxyl group of carbon C9, yielding 9-*O*-Ac-Sia^7^.

Sialic acids further modified by *O*-acetylation are expressed in a tissue and cell-specific manner on glycoproteins or glycolipids on normal cells, and those modified Sias can act as selective receptors for entry of some viruses^8^. However, abnormal expression of sialic acid-containing glycoconjugates and *O*-acetylated derivatives has also been noted to correlate with tumorigenesis. For example, the ganglioside GD2 is a tumor-specific marker for neuroblastoma, and antibodies against it are currently part of maintenance therapy for this pediatric malignancy^9^. Interestingly, the 9-*O*-acetylated form of GD2 may be a more specific treatment target than GD2^10^. The ganglioside GD3 and its 9-*O*-acetylated form (9-*O*-Ac-GD3, also called CD60b) are sialylated glycolipids expressed in glioblastomas^11^ and melanomas^12^. In GD3 (Sia-α2,8-Sia-α2,3-Gal-β1,4-Glc-β1,1′-Cer), the terminal Sia is α2,8-linked and the *O*-acetylation only occurs on the terminal Sia residue.

We previously reported expression of 9-*O*-Ac-Sia on pre-B ALL cells using antibodies against 9-*O*-Ac-GD3 and a lectin from *Cancer antennarius* (CCA lectin). Moreover, we showed that its cell surface expression correlates with both drug resistance in a tissue co-culture model in the presence of stromal protection, and with cell survival in mice^3^. CCA lectin binds to both sialoglycoproteins and gangliosides when these are 9-*O*-acetylated^13,14^, and thus we were not able to determine unambiguously if pre-B ALL cells express 9-*O*-Ac-glycoproteins. However, we inferred their presence based on the finding that CCA lectin still bound to the surface of cells lacking a functional ST8siaI, the only sialyltransferase known to be able to generate GD3 from GM3.

The current study was undertaken to identify such sialoglycoproteins. We here report that nucleolin (NCL) is expressed on the surface of pre-B ALL cells as a sialoglycoprotein which is modified by 9-*O*-acetylation. NCL has no enzymatic function and can be best described as “multi-functional”^15,16^, in the sense that it can have multiple subcellular locations (nucleus/nucleolus, cytoplasm and plasma membrane), bind to multiple biomolecules (RNA, other proteins)^17,18^, and has been implicated in a variety of biological processes including angiogenesis, migration, apoptosis^19,20^ and regulation of cell size^21^. Increased NCL expression is associated with worse prognosis of, among others, acute myeloid leukemia^22^, chronic lymphocytic leukemia^23^, diffuse large B-cell lymphoma^24^ and pancreatic ductal cancer^25^. Interestingly, NCL is viewed as a target for cancer therapy, and our studies further show that some pre-B ALL cells are sensitive to anti-NCL treatment using AS1411, a DNA aptamer that specifically binds NCL.

## Results

### NCL identified by CCA lectin column chromatography is widely expressed in pre-B ALL

**Figure 1:**
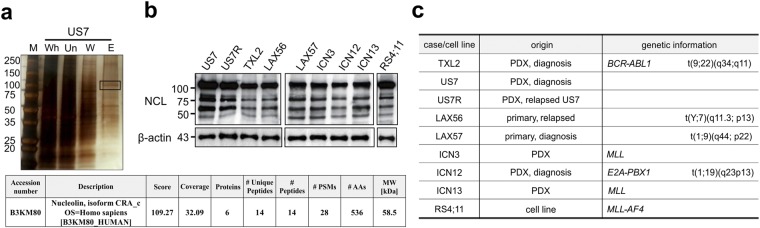
CCA lectin chromatography identifies a 100-kDa protein in pre-B ALL cells as NCL. (**a**) Image of silver-stained SDS-PAA gel showing different column fractions of a US7 pre-B ALL cell lysate. M = marker; Wh = whole cell lysate; Un = flow-through from CCA lectin affinity column; W = wash; E = eluate. The boxed area indicates the band analyzed by mass spectrometry. Table: Characteristics of 100 kDa NCL band analyzed by mass spectrometry (the 58.5 kDa CRA_c nucleolin isoform has 100% sequence identity with the 75 kDa CRA_b isoform). (**b**,**c**) Western blot analysis (**b**) of different pre-B ALLs as indicated in (**c**) using antibody against NCL. PDX, patient-derived xenograft.

To identify *O-*acetylated sialoglycoproteins in pre-B ALL cells, we applied a total cell lysate of US7, a human patient-derived pre-B ALL, to a CCA lectin affinity column. A silver stained gel of the elution fraction showed major bands including proteins of around 50 and 100 kDa (Fig. 1a, top). The elution pattern of a lysate of a different pre-B ALL TXL2 was similar (not shown). Proteomic analysis subsequently identified NCL as a main component of approximately 100 kDa (hereafter referred as 100 kDa) binding to the CCA lectin (Fig. 1a table).

We next examined representatives of different subcategories of pre-B ALL to determine if NCL expression varies in such samples, using total cell lysates and Western blotting (Fig. 1b,c). NCL was detected at comparable levels as a ≈100 kDa and a 75 kDa core protein in all samples. Smaller products representing isoforms or proteolytic products were also detected. In agreement with this, meta-analysis of large gene expression array data sets including almost 500 pre-B ALLs also showed high expression levels of *NCL* mRNA (Supplementary Fig. S1).

### NCL is a 9*-O-*acetylated sialoglycoprotein

**Figure 2:**
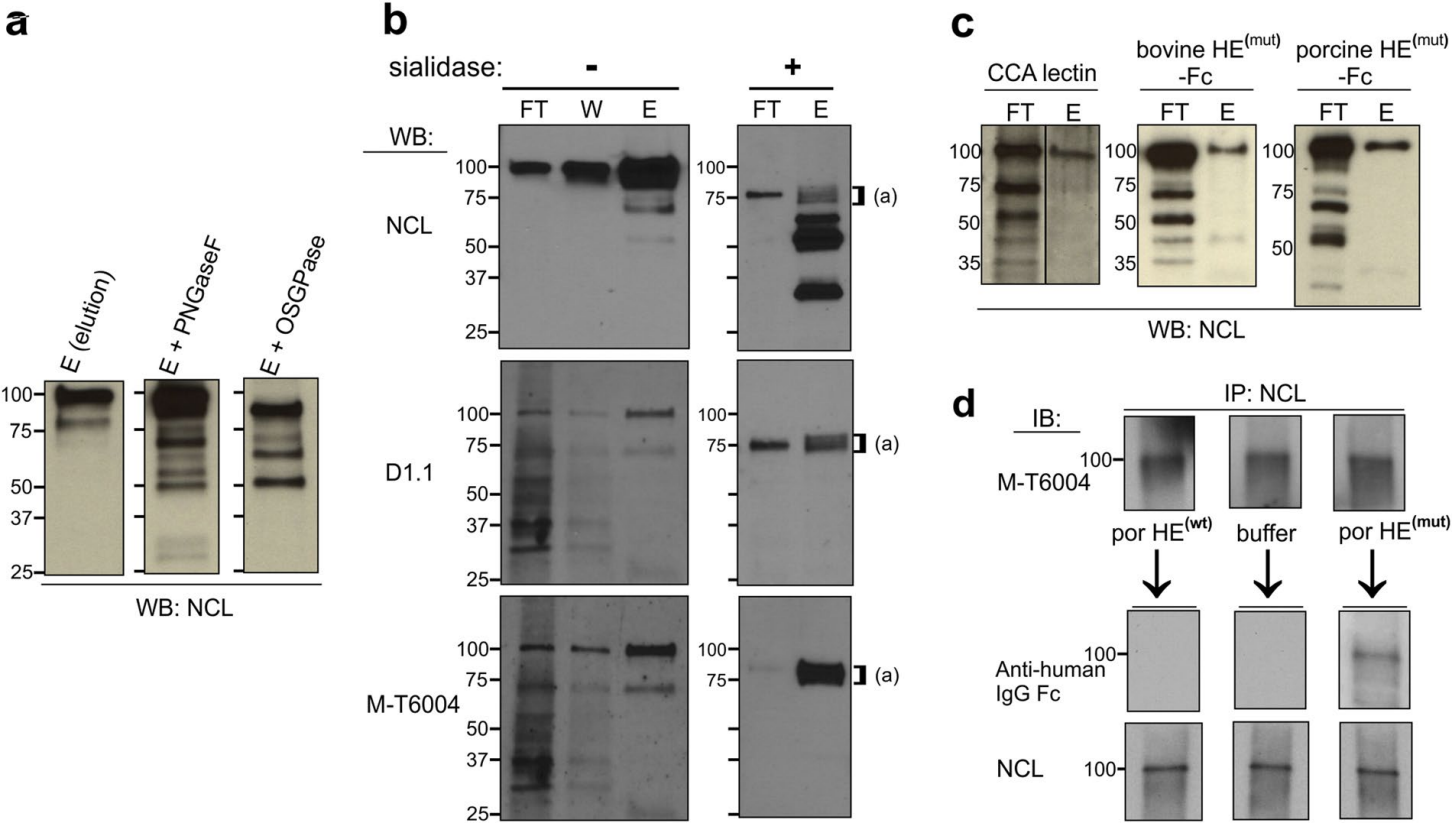
NCL is sialylated and *O-*acetylated in pre-B ALL. (**a**) Treatment of CCA lectin column elution fractions of US7 cells with the indicated glycosidases followed by Western blotting using anti-NCL antibodies. (**b**) Analysis of CCA lectin column fractions of US7 lysates treated with buffer (−) or *C. perfringens* sialidase (+) using the antibodies indicated to the left. FT = flow-through; W = wash; E = eluate. Blots were stripped and consecutively reacted. (**c**) Binding of NCL to different *O-*Ac-Sia-recognizing lectins as indicated. (**d**) Immunoprecipitated (IP) NCL was run in triplicate on SDS-PAA gels. After a control reaction with CDw60 (M-T6004) antibody to ensure the presence of *O-*Ac-Sia, membranes were stripped and treated with porcine torovirus HE^(wt)^ -Fc, buffer, or porcine HE^(mut)^ -Fc, then probed with an anti-human IgG antibody that will detect HE-Fc lectins, and with NCL (Abcam) antibody as indicated. All panels, lysates of US7 pre-B ALL cells.

To further analyze the NCL that carries 9-*O*-acetylated sialoglycans in pre-B ALL, we treated the fraction of US7 cell lysates that had been eluted from a CCA lectin column with peptide N-glycosidase F (PNGase F) or with *O-*sialoglycoprotease (OSGPase). Treatment with PNGase F, which only cleaves N-linked glycans, replaced the 100 kDa NCL band with a 90–95 kDa band, indicating that CCA lectin-binding NCL contains N-linked glycans (Fig. 2a). We additionally analyzed binding of NCL to the lectin SNA (*Sambucus Nigra*), which is selective for detection of α2, 6-linked sialic acids which can be attached to N-linked glycans. The 100 kDa NCL band was found in the flow-through as well as in the elution fraction, indicating that Sia is attached via an α2,6 linkage on only some of the NCL (Supplementary Fig. S2). The 100 kDa NCL glycoforms captured by the CCA lectin column were reduced in size by treatment with OSGPase (Fig. 2a). This enzyme digests proteins with clustered patches of *O*-linked sialoglycans^26^ such as present on sialomucins^27,28^. Interestingly, treatment of CCA lectin column fractions with *C. perfringens* sialidase reduced the molecular mass of both the flow-through (non *O-*Ac-Sia) as well as the entire 100 kDa CCA lectin-binding (*O-*Ac-Sia) NCL fraction to around 75 kDa (Fig. 2b, right panel). This suggests that the 100 kDa NCL glycoforms are all sialylated. Proteolytic products smaller than full-length NCL were also seen (Fig. 2b). We confirmed that most of the 100 kDa NCL is sialylated by treatment of an unfractionated RIPA buffer cell lysate with sialidase (Supplementary Fig. S2).

Numerous monoclonal antibodies (mAbs) are available that specifically recognize 9*-O-*Ac-GD3. However, it is not clear if they exclusively react with gangliosides. We found that both D1.1 and M-T6004 mAbs generated against *O-*Ac-GD3 also detected numerous proteins on a Western blot (i.e., flow-through lanes Fig. 2b left panels). Interestingly, the antibodies specifically reacted with a limited number of proteins, one of 100 kDa, as well as a 75 kDa protein captured by the CCA lectin column (lane “E”). Upon sialidase treatment, the 100 kDa band disappeared and both antibodies detected a doublet of around 75 kDa (Fig. 2b right panels).

To further support the finding that NCL is a 9*-O-*acetylated sialoglycoprotein, we tested two nidovirus lectins that recognize 9*-O-*Ac-Sia^29,30^. The recombinant porcine torovirus HE^(mut)^ -lectin primarily detects 9-mono*-O-*Ac modified sialic acids whereas the bovine HE^(mut**)**^ -lectin also detects 7,9-di*-O-*Ac-Sia. Both are largely insensitive to linkage of Sia (α2,3 or α2,6)^30^. The esterase moieties of these probes were mutated to inactivate their 9*-O-*acetyl esterase activities and they are tagged by fusion to human IgG1-Fc. Similar to the CCA lectin affinity column, columns with both porcine torovirus and bovine coronavirus HE-Fc retained the 100 kDa NCL protein (Fig. 2c). We also immunoprecipitated NCL from a lysate of US7 cells. Reaction of the membranes with a NCL antibody confirmed its immunoprecipitation and retention on the membrane (Fig. 2d, lower panel). The M-T6004 antibody detected NCL (Fig. 2d upper panel). The triplicate Western blots were then incubated with either porcine HE^(wt)^ -Fc or porcine HE^(mut)^ -Fc, or control buffer. The HE^(wt)^ -Fc as an active sialate 9*-O-*acetyl esterase is expected to remove 9*-O-*Ac from the sialic acids on NCL. Indeed, as shown in Fig. 2d (middle panel), the lectin part of the HE protein was no longer able to bind to NCL after the esterase moiety had cleaved the 9*-O-*Ac group. Binding of the HE^(mut)^ -Fc to non-treated NCL was detected as expected.

### The 100 kDa NCL glycoform is expressed on the surface of pre-B ALL cells

**Figure 3:**
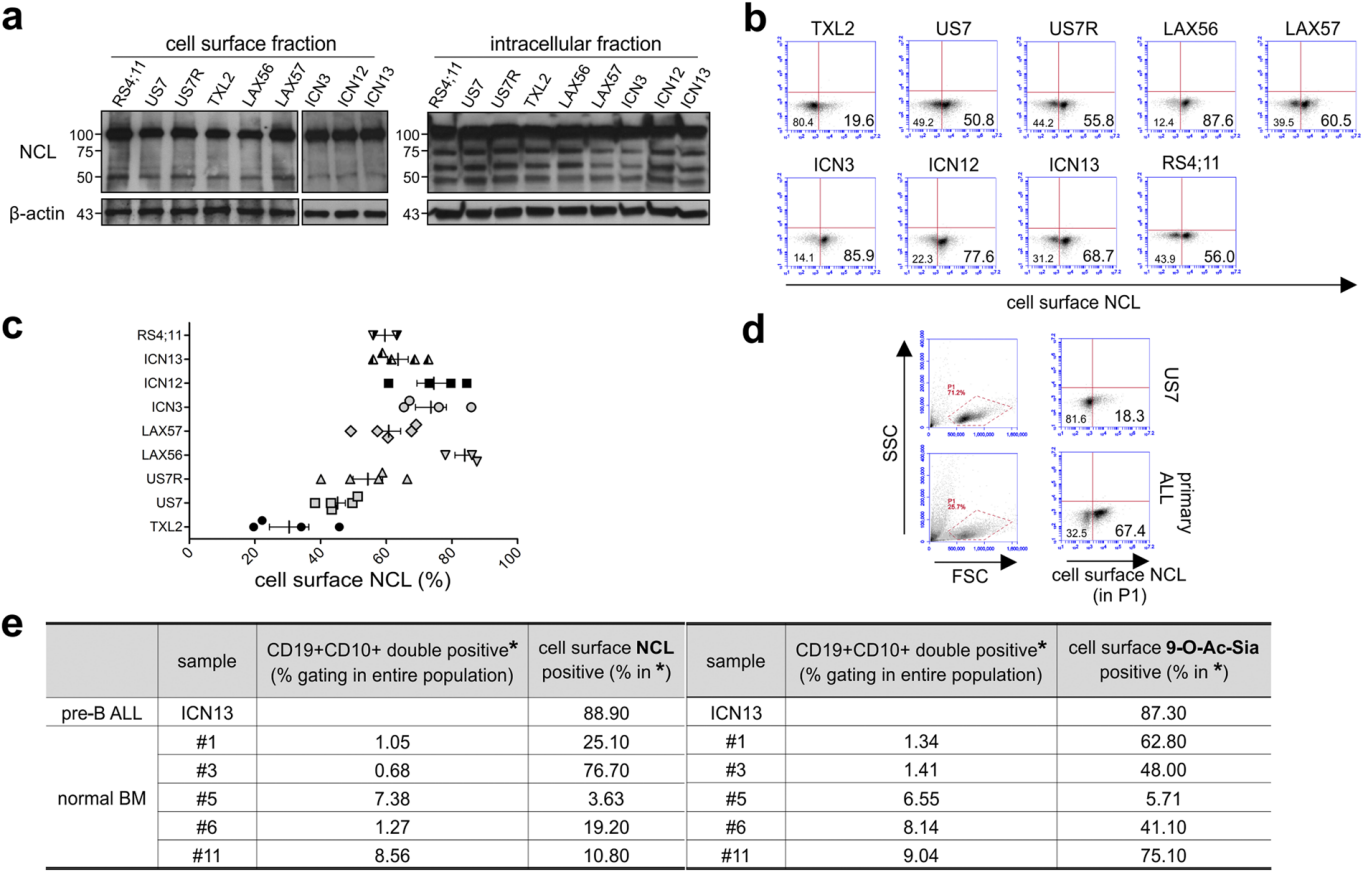
The 100 kDa NCL glycoform is expressed on the surface of pre-B ALL cells. Analysis of the indicated pre-B ALLs using NCL antibody. (**a**) Western blotting on cell surface (biotinylated) and intracellular (non-biotinylated) fractions. Note: Different WB membranes of the two fractions; the WB of the intracellular fraction was exposed shorter than that of the cell surface fraction. (**b**–**e**) Flow cytometry. (**b**) Representative analysis of cell surface NCL in different pre-B ALLs. (**c**) Summary graph of n = 2–5 independent experiments with the indicated pre-B ALLs. (**d**) NCL expression in gate P1 on primary fresh pre-B ALL PB sample containing >95% blasts. For FACS, cells were fixed without permeabilization. (**e**) Analysis for cell surface NCL and 9-*O*-Ac-Sia on normal pre-B cells. Percentage of cells in five different normal bone marrow samples, processed as described in Materials and Methods, that were positive for cell surface NCL and 9-*O*-Ac-Sia in the CD19+, CD10+ gate (gating strategy, see Supplementary Fig. S3). ICN13, positive control run in the same experiment.

The identification of NCL as a sialoglycoprotein is important, as Losfeld *et al*.^31^ demonstrated that glycosylation is needed for the expression of NCL on the cell surface. Moreover, Bates *et al*.^32^ hypothesized that normal cells only transiently express NCL outside the nucleus, in response to specific stimuli, whereas cancer cells would hijack this and express non-nuclear NCL constitutively. This places a specific significance on glycosylated NCL at the plasma membrane of cancer cells, where it can engage and transport a variety of cargos. We therefore examined cell surface NCL on pre-B ALL cells in more detail. Based on Western blotting (Fig. 3a, compare left and right panels), we confirmed that the 75 kDa NCL core protein was exclusively detected in the cytoplasmic and not in the membrane fraction. Cell surface NCL had an apparent molecular mass of 100 kDa and the non-biotinylated, intracellular protein fraction also contained 100 kDa NCL protein, which could represent glycosylated NCL in different endocytic compartments.

We used flow cytometry to quantitate the percentage of pre-B ALL cells expressing NCL on the cell surface. NCL has a very rapid turnover on the plasma membrane^33^ and because of this, cells were fixed before antibody staining to detect it at this location. Our analysis showed that each pre-B ALL population had a different percentage of cells with NCL on the surface (Fig. 3b). For example, TXL2 had the lowest percentage, whereas in other pre-B ALL populations such as LAX56, more than 80% of cells were positive. The mean fluorescent intensity (MFI), as a measure of the number of NCL molecules per cell, was comparable among the different pre-B ALLs (not shown), indicating similar levels of NCL expression on those cells that did express it. Interestingly, all cultures seemed to contain discrete populations that either expressed or did not express cell surface NCL. The percentage of cells positive for cell surface NCL appeared to be a relatively stable property of each individual pre-B ALL, as sampling of the cells on multiple occasions gave a consistent result (Fig. 3c). In agreement with the results of Western blotting for intracellular NCL (Fig. 3a) after an additional permeabilization step to also detect intracellular NCL, all pre-B ALLs were almost 100% positive for NCL (not shown).

To determine whether tissue culture affects NCL expression on pre-B ALL cells, we also assessed NCL on two primary, viably frozen bone marrow (BM) and one fresh peripheral blood (PB) pre-B ALL patient sample. Compared to US7 run in the same experiment, non-cultured pre-B ALL cells from a primary PB sample showed a high percentage of cell surface NCL (Fig. 3d). The MFI, as a measure of the number of NCL molecules per cell, was comparable to that of cultured pre-B ALLs, supporting the concept that pre-B ALL populations mainly differ in the percentage of cells that express NCL and that different populations have comparable expression levels on each cell. In two pre-B ALL BM samples, 87.7 and 41.2% of the cells were positive for cell surface NCL (not shown). We also evaluated cell surface NCL on normal (non-leukemic) human pre-B cells. These are characterized by positivity for CD19 and CD10. ICN13 was included as a positive control. As summarized in Fig. 3e and Supplementary Fig. S3, the percentage of normal pre-B cells expressing surface NCL varied between different donor samples.

### NCL on the cell surface partly co-localizes with total 9*-O-*Ac-Sia

**Figure 4:**
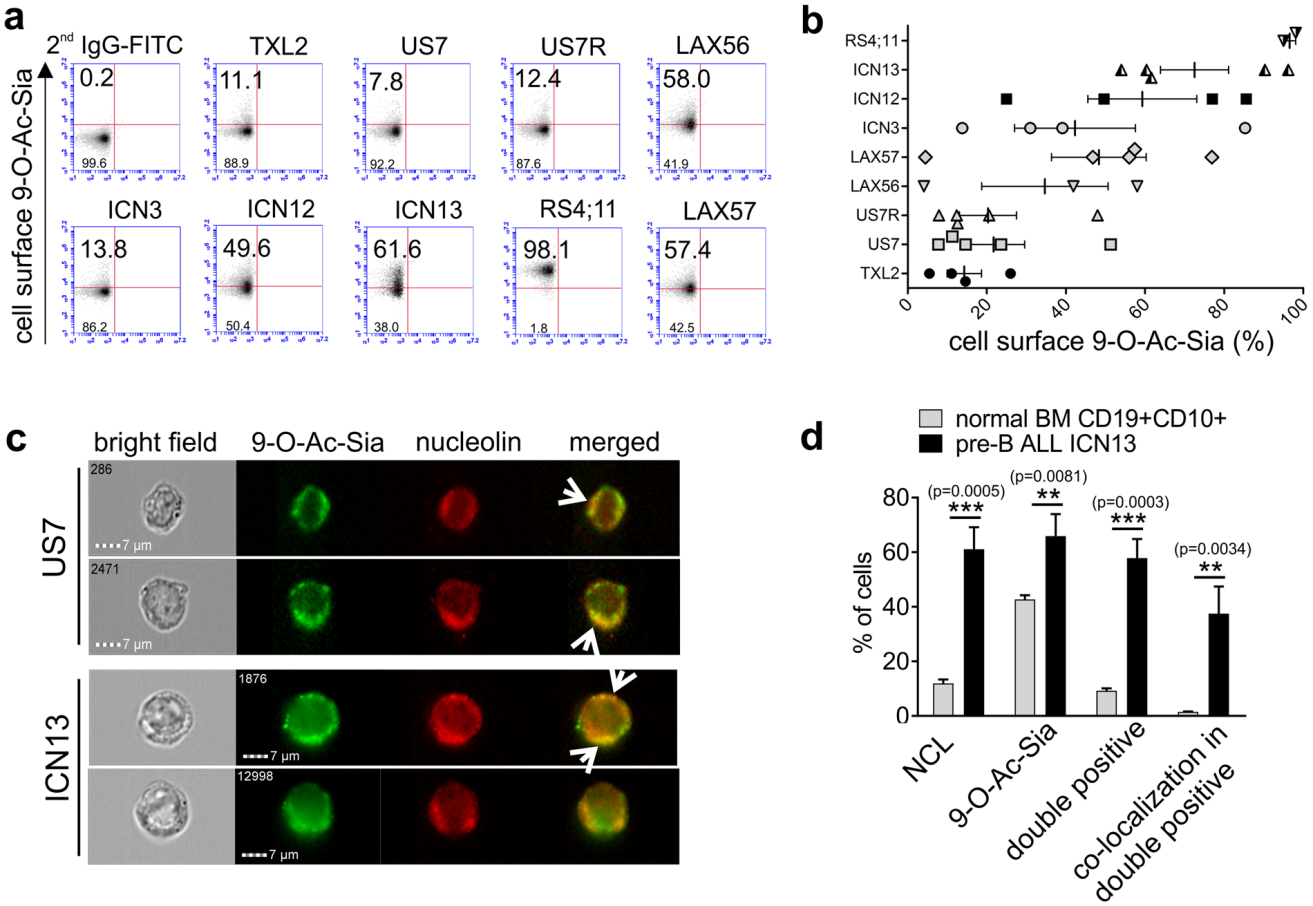
NCL and 9-*O*-acetylated sialoglycoconjugates are co-expressed on the surface of pre-B ALL cells. (**a**) Different pre-B ALLs as indicated were analyzed for cell surface 9-*O*-Ac-Sia using bovine coronavirus or porcine torovirus HE^(mut)^ –Fc as detection lectin. Numbers indicate the percentage of positive cells. The signal of control IgG-FITC was used to determine the gate. Representative experiment with simultaneous analysis of all samples; in total 4–5 independent experiments. (**b**) Summary graph of FACS analysis in different experiments. (**c**) Representative ImageStream images of US7 and ICN13 pre-B ALL cells. Left images show bright field. The right panels show the merged images of 9-*O*-Ac-Sia signal (green) and NCL (red). Arrows point to locations where the signals overlap. Magnification, x600. (**d**) Summary quantification of ImageStream analysis on normal human CD19+ CD10+ bone marrow cells and ICN13 pre-B ALL cells indicating percentages of cells in each category. Triplicate samples from one cell population, stained and processed separately. Values: mean ± S.E. Unpaired t-test, two-tailed p-value.

Different cancer cell types express a characteristic, but varying percentage, of cell surface *O-*acetylated sialoglycoproteins that can be detected by the specific viral HE-Fc lectins^30,34^. We next measured cell surface 9*-O-*Ac-Sia on pre-B ALL cells using FACS, the HE-Fc lectins and anti-human IgG Fc-FITC secondary antibodies. As shown in Fig. 4a, there was a large difference among pre-B ALLs in the percentage of cells positive for surface 9*-O-*Ac-Sia, ranging from nearly all cells (RS4;11) to only about 8% of the cells (US7). When the same pre-B ALL was examined in several independent experiments, a large variation in the percentage of positive cells was measured (Fig. 4b). Interestingly, pre-B ALL samples highly positive for surface 9*-O-*Ac-Sia tended to also be highly positive for cell surface NCL (compare Fig. 3c with Fig. 4b). The co-localization of the 9*-O-*Ac-Sia signal with that of NCL was further investigated using ImageStream flow cytometry (Fig. 4c). The 9*-O-*Ac-Sia signal appeared as punctate structures on the surface of both US7 and ICN13 pre-B ALL cells. Part of NCL signal was also concentrated in punctate structures, and co-localization of NCL and 9*-O-*Ac-Sia was evident on some areas of the plasma membrane (Fig. 4c, arrows, merged). Of the ≈4000 cells analyzed, 36.6% were clearly positive for both NCL and 9*-O-*Ac-Sia, with a small percentage of cells singly positive for only NCL (3%) or only for 9*-O-*Ac-Sia (4%). Their location was also compared on normal human bone marrow CD19+ CD10+ pre-B precursors. Consistent with Fig. 3e, Fig. 4d shows that fewer normal precursor B cells expressed cell surface NCL than leukemic pre-B cells. Moreover, on average only around 10% of normal pre-B cells expressed both NCL and 9-*O-*Ac-Sia on the same cell, compared to around 60% for ICN13 pre-B ALL cells. Interestingly, whereas around 37% of ICN13 pre-B ALL cells that were positive for both NCL and 9-*O-*Ac-Sia had a co-localization of the two signals, this was the case in only 1.5% of the normal double positive pre-B cells. Taken together, this suggests that NCL on normal pre-B cells contains less 9-*O*-Ac-Sia.

**Figure 5:**
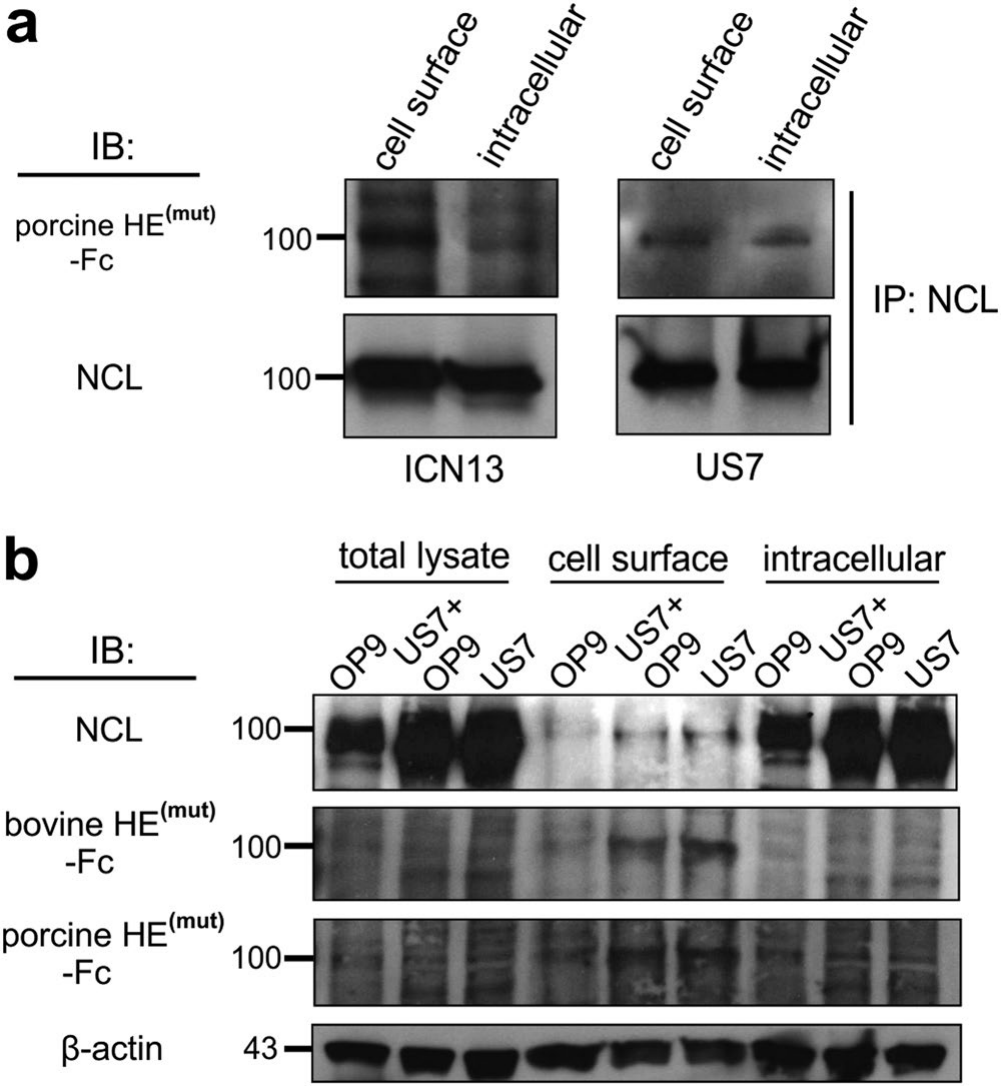
Surface NCL contains more 9*-O-*Ac-Sia than cytoplasmic 100 kDa NCL. (**a**) Quantitative comparison of 9*-O-*Ac-Sia-containing NCL immunoprecipitated from an equal cell equivalent of cell surface and intracellular fractions. (**b**) Western blot analysis using the detection reagents indicated to the left on total cell lysates, cell surface proteins isolated by live cell biotinylation and streptavidin-column isolation and their intracellular fractions. Note: equal amounts of protein lysate were loaded in each lane. Blots were sequentially stripped and re-probed.

To directly compare the levels of 9*-O-*Ac-Sia on NCL located at the cell surface and intracellularly in leukemia cells, we biotinylated cell surface proteins on US7 and ICN13 cells as before. Similar amounts of immunoprecipitated NCL from cell surface and intracellular fraction were then compared (Fig. 5a bottom panel, NCL Western blot) for 9*-O-*Ac-Sia using the porcine HE^(mut)^ as a detection probe. The signal for 9*-O-*Ac-Sia in the lanes with cell surface NCL was stronger than that in the lanes with intracellular NCL, indicating that cell surface sialylated NCL is preferentially 9*-O-*acetylated compared to intracellular NCL (Fig. 5a top panels).

We use OP9 stromal cells to support long-term growth and viability of pre-B ALL cells^35–37^. As cell surface NCL levels were also reported to correlate with cell stimulation and growth (e.g.,^38^) we used FACS to compare NCL levels on pre-B ALL cells that were held under circumstances that do not promote cell growth. However, cell surface NCL levels were not reproducibly different on pre-B ALL cells grown with OP9 stroma in complete growth medium, on pre-B ALL cells that were incubated in complete medium but without OP9 stroma for up to 72 hr, and on pre-B ALL cells that were kept overnight in culture medium without FBS (data not shown). We also analyzed cell surface and cytoplasmic NCL in pre-B ALL cells with or without OP9 stromal support, using Western blotting. As shown in Fig. 5b, stromal OP9 cells alone contained comparatively low levels of NCL. Whether pre-B ALL cells were kept in suspension or were adhered to the OP9 cells had little effect on their NCL total levels, 9*-O-*acetyl sialylation of NCL or its subcellular distribution (Fig. 5b). Per µg of lysate, more NCL was present in the cytosolic than in the membrane fraction yet the signals from both porcine HE^(mut**)**^ -Fc and bovine HE^(mut**)**^ -Fc detecting the 100 kDa NCL were stronger in the cell surface fraction than in the intracellular fraction. Taken together, these results indicate that cell surface NCL carries more 9*-O-*Ac-Sia than intracellular 100 kDa NCL.

### Cell surface NCL is increased by drug treatment, lipid raft disruption, and sialic acid removal

**Figure 6:**
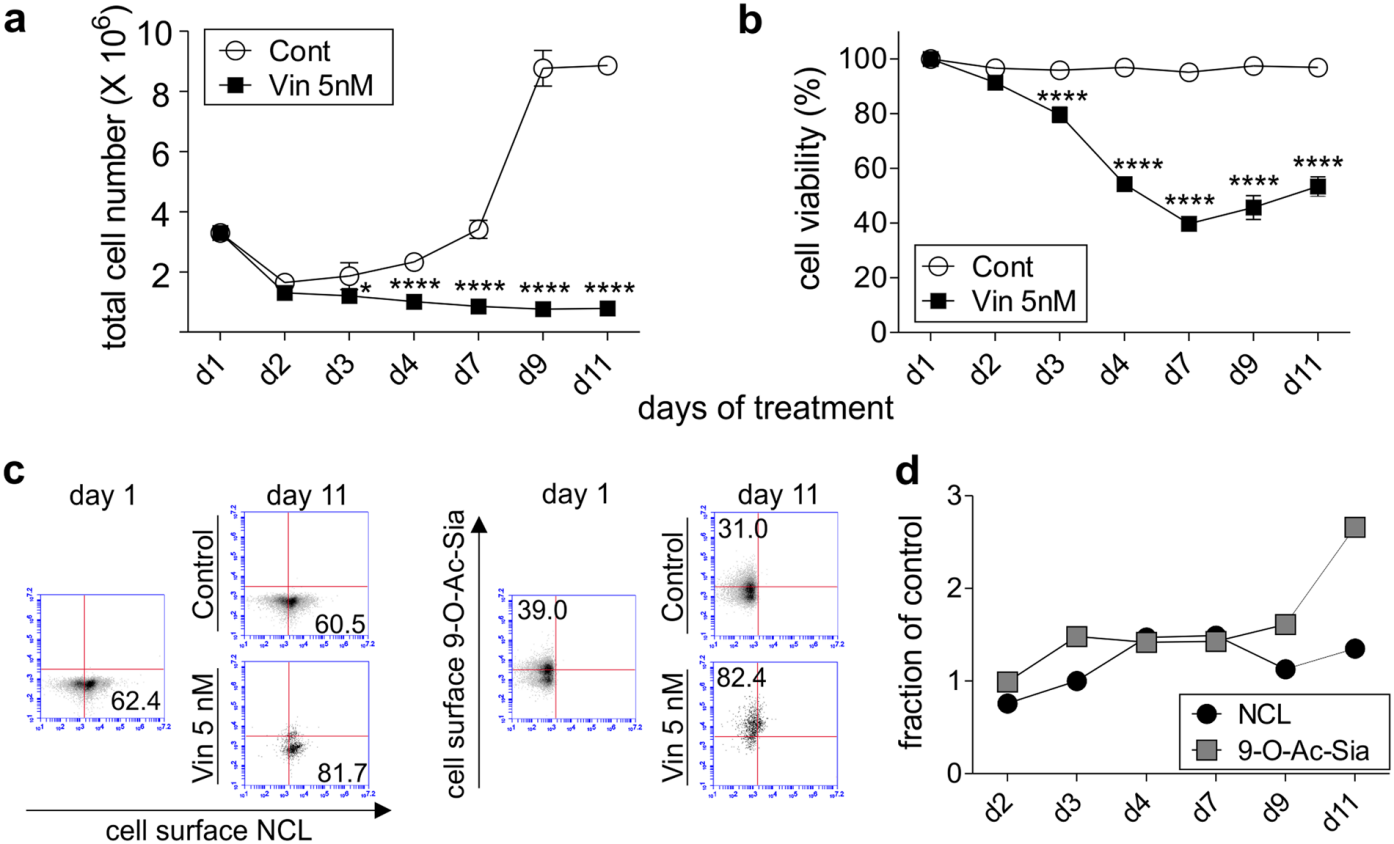
Chemotherapeutic drug treatment increases NCL and 9*-O-*Ac-Sia on the surface of pre-B ALL cells. Long-term drug treatment of US7 pre-B ALL cells with 5 nM vincristine or DPBS while cells are protected by stromal support. (**a**) total cell number, (**b**) viability. Graphs report mean ± S.E. ^*^p = 0.0139; ^****^p < 0.0001 (vincristine treatment versus control on each time point). Two-way ANOVA, Sidak’s multiple comparisons test. (**c**,**d**) Analysis of cells positive for cell surface NCL or 9*-O-*Ac-Sia upon vincristine treatment compared to parallel non-vincristine treated cultures sampled at the same time points. (**c**) Representative FACS plots. Controls: isotype control for NCL; IgG-FITC secondary antibody only for porcine HE^(mut)^ -Fc. (**d**) Graphical representation showing the fraction of cells positive for the indicated structures under vincristine/non-vincristine treatment. One of three experiments with similar results.

We also performed a time course in which US7 pre-B ALL cells were treated with the chemotherapeutic drug vincristine in the presence of stromal support. Such treatment typically resulted in the persistence of a small number of pre-B ALL cells that were unable to proliferate (Fig. 6a) but survived in the presence of drug, as measured by an increased percentage of live (Trypan-blue excluding) cells in the total remaining population (Fig. 6b). As illustrated in Fig. 6c, we detected increased cell surface NCL on the pre-B ALL cells that survived. We previously showed that pre-B ALL cells surviving chemotherapy have increased cell surface 9*-O-*Ac-GD3 and 9*-O-*acetylated α2,3 or α2,6-linked sialoglycoproteins as reported by CCA lectin^3^. Consistent with this, we here also measured increased levels of 9*-O-*Ac-Sia on the pre-B ALL cells using the porcine HE^(mut)^ -Fc. Interestingly, NCL expression on the surface clearly did not correlate with cell division, as cell proliferation was rapidly inhibited by vincristine, but cell surface NCL persisted and even increased over time (Fig. 6d).

**Figure 7:**
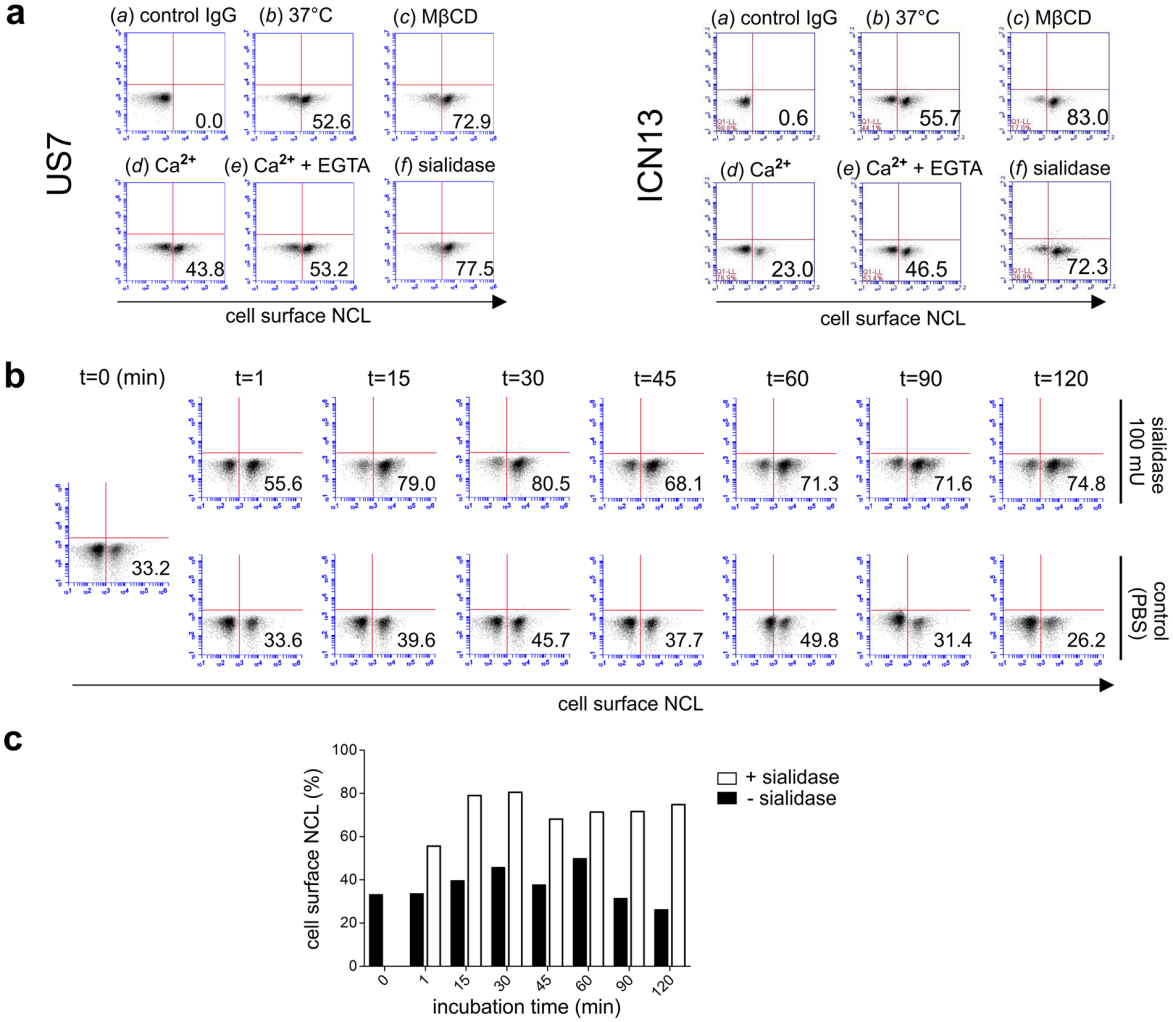
Retention of NCL on the cell surface of pre-B ALL cells is regulated by lipid raft presence and sialylation. (**a**) Analysis of cell surface retention of NCL by FACS with anti-nucleolin antibodies. US7 (left panel) and ICN13 (right panel) pre-B ALL cells treated with (*a*) isotype IgG control, (*b*) no treatment (buffer) and treatment for 1 hr at 37 °C with (*c*) 100 mU sialidase, (*d*) 5 mM MβCD, (*e*) 1 mM CaCl_2_, and (*f*) 1 mM CaCl_2_ + 5 mM EGTA. (**b**,**c**) FACS plots (**b**) and graphical representation of results (**c**) of time course of US7 cell treatment with sialidase. Numbers in the lower right indicate the percentage of positive cells. (**a**) representative of 3–4 independent experiments, (**b**,**c**) one of 2 individual experiments.

To investigate regulation of NCL on the surface of pre-B ALL cells, we treated these cells with cholesterol-depleting methyl β-cyclodextrin (MβCD), which was reported to cause redistribution of cell surface NCL from lipid rafts to non-lipid rafts^39^. We also treated the cells with external CaCl_2_, which induces endocytosis of cell surface NCL^20,39^. As shown in Fig. 7a, compared to controls, MβCD treatment correlated with a significant increase in cell surface NCL on US7 and ICN13, and treatment with Ca^2+^ reduced NCL on the cell surface. Treatment of these pre-B ALLs with bovine HE^(wt)^-Fc, which encodes an active 9-*O*-Ac-esterase, was associated with a small increase (around 4%-10% in 3 experiments) in cell surface NCL detection compared to control cells (not shown).

We had detected glycosylated 100 kDa NCL both intracellularly and on the cell surface using Western blots. To specifically address the effect of sialylation on the pool of NCL at the cell surface, we treated living US7 and ICN13 pre-B ALL cells with *C. perfringens* sialidase, which will only remove Sia from NCL exposed on the cell surface. As shown in Fig. 7a, there was a marked increase in cell surface NCL detected by FACS (US7 from 52.6% to 77.5% and ICN13 from 55.7% to 72.3%) after enzymatic removal of Sia.

The increase of cell surface levels of NCL by sialidase treatment was unexpected. To investigate this in more detail, a time course of treatment was carried out. Controls were held in PBS at 37 °C without sialidase. As shown in Fig. 7b (and graphical representation in Fig. 7c), even a one-minute incubation (t = 1 min) with sialidase increased the percentage of cells that were positive for cell surface NCL compared to the control. The maximum percentage of pre-B ALL cells positive for cell surface NCL even increased to 80.5% at t = 30 min.

We also tested the effects of these treatments on cell surface 7,9-di*-O-*Ac-Sia as reported by the bovine HE^(mut)^-Fc and measured by FACS (Supplementary Fig. S4). The levels of these structures on the cell surface were not measurably regulated by Ca^2+^ or MβCD treatment. As expected, HE^(wt**)**^ -Fc destroyed the binding ability of the 7,9-di*-O-*Ac-Sia-recognizing lectin part, and sialidase treatment had a similar effect.

**Figure 8:**
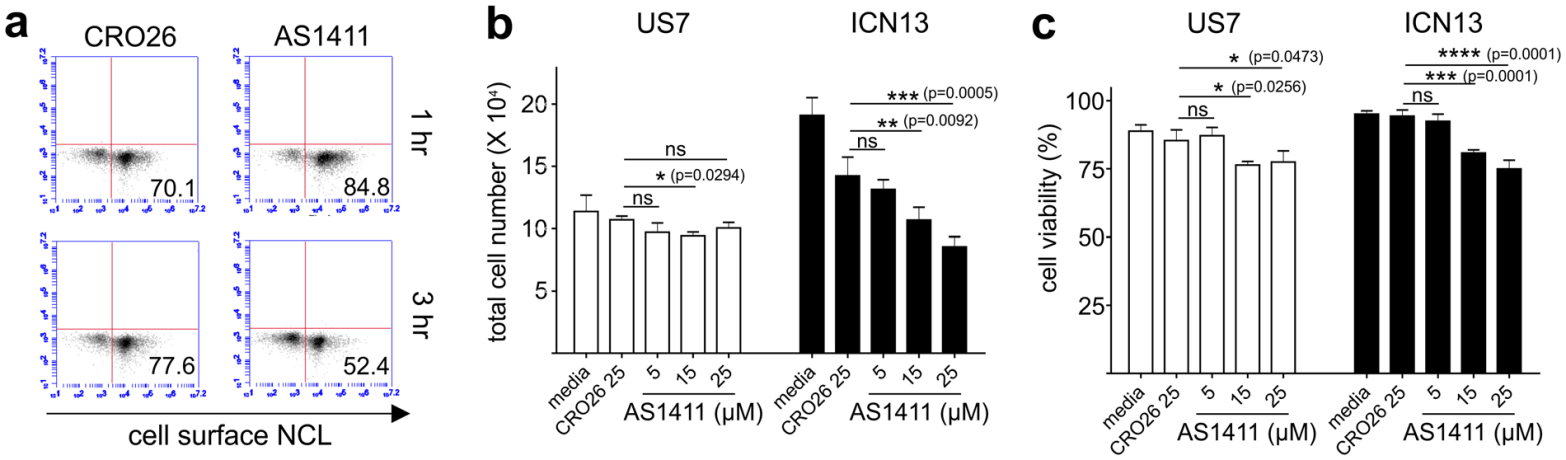
Targeting of NCL with the AS1411 aptamer inhibits proliferation of pre-B ALL cells. (**a**) FACS analysis for cell surface NCL in ICN13 cells treated with 15 µM AS1411 or control CRO26. (**b**,**c**) Treatment with the indicated concentrations of AS1411 or control CRO26 aptamers for 48 hr in the absence of stroma. (**b**) total cell number, (**c**) viability. One experiment of two with different sources of aptamers. Values- mean ± S.E. n.s., not significant, ^*^p < 0.05, ^**^p < 0.01, ^***^p < 0.001. One-way ANOVA.

To investigate if NCL could be a therapeutic target in pre-B ALL, we treated leukemia cells with a widely-studied NCL-specific aptamer AS1411 and control CRO26^32,40^. Interestingly, after a 3 hr treatment with 15 µM AS1411 but not with CRO26, cell surface NCL decreased on ICN13 cells (Fig. 8a). When ICN13 cells were treated for 48–72 hr with AS1411, cell proliferation and viability were significantly inhibited compared to control CRO26. However, the same concentration of AS1411 had a much smaller effect on US7 cells (Fig. 8b,c). Treatment of other pre-B ALLs with AS1411 similarly showed a differential sensitivity to NCL inhibition (Supplementary Fig. S5). TXL2 did not respond to treatment with AS1411, which inhibited cell division but did not cause cell death in ICN3. Proliferation of RS4;11, similar to ICN13, was inhibited by AS1411. AS1411 had the strongest effect on LAX56 and LAX57, in which both cytostatic and cytotoxic effects were measured.

## Discussion

We identified NCL as a sialoglycoprotein modified by 9-*O*-acetylation in pre-B ALL. NCL has not been studied in pre-B ALL although it is viewed as a possible target for treatment in other cancers. In this context, the subcellular location and distribution of NCL protein appear to be key factors, seeing that normal cells were reported to express NCL mainly in the nucleus whereas some cancer cells constitutively express NCL on the cell surface^32,33^. Thus the question of what regulates expression of NCL on the surface of cells is of significant interest. Hovanessian *et al*.^33^ showed that NCL needs to be glycosylated to appear on the surface, indicating that cancer cells that express high levels of cell surface NCL must also maintain high glycosylation levels on NCL. As Hovanessian *et al*.^33^ also reported that the half-life of cytoplasmic and cell surface NCL in carcinomas is only around 45 minutes, cancer cells either recycle NCL very efficiently, with minimal deglycosylation, or rapidly synthesize and glycosylate it *de novo*. Regardless of the exact mechanism, we found that in pre-B ALL cells, only the 100 kDa glycosylated NCL and not the core 75 kDa NCL is located on the surface, which is in agreement with other reports^31^.

Previously, seven potential glycosylation sites were identified in NCL. These include five *O-*linked glycosylation sites in the N-terminal end of NCL, and two N-linked sites N317 and N492, in T-ALL Jurkat cells^41,42^. We found that most, if not all of the 100 kDa NCL glycoprotein band contains Sia, and that the Sia in NCL can be terminal on both the N- and the *O*-linked glycans. Because the ≈100 kDa NCL glycoform bound by CCA lectin was completely cleaved by OSGPase, all 9*-O-*Ac-Sia-modified NCL contains clustered *O*-linked sialic acid, probably on glycans attached to T84, T92, T105, T106, and T113 in the N-terminal part of NCL^41,42^. Finally, the finding that antibodies raised against 9*-O-*Ac-GD3 cross-react with sialylated NCL, as seen here, suggests that NCL could contain disialic acid (Sia-2,8-Sia-) structures. Although not common, these have been reported on glycoproteins other than NCAM including the leukocyte common antigen CD45^34^. In summary, we conclude that all NCL on the surface of these pre-B ALL cells is sialylated, sialylation is found both on N- and *O*-linked glycans, and all 9-*O*-acetylated NCL sialoglycoforms contain clustered *O*-linked glycans.

In previous studies, others and we identified 9*-O-*Ac-GD3 on the surface of pre-B ALL cells based on the use of antibodies which have also been widely used for this purpose in immunohistochemistry. Fox *et al*.^43^ reported that UM4D4 monoclonal antibodies which recognize 9*-O-*Ac-GD3 actually detect sialoglycoproteins of 94–70 kDa Mw in T cells. The Jones antibody, which reacts with 9*-O-*Ac-GD3, also detected glycoproteins, in the 100 kDa and 37 kDa range^44^. In our studies, D1.1 and M-T6004 mAbs, which are sold as antibodies against 9*-O-*Ac-GD3, detect glycoproteins, and both reacted with 100 and 70 kDa proteins binding to CCA lectin. As immunoprecipitated NCL reacts with M-T6004, we conclude that NCL is one of the principle *O-*acetylated sialoglycoproteins detected by M-T6004. We cannot exclude the possibility that both D1.1 and M-T6004 recognize additional *O-*acetylated sialoglycoproteins in the 75 kDa range that are not sensitive to sialic acid removal by treatment with *C. perfringens* sialidase, which prefers α2,3-linked Sia and may be inhibited by 9*-O-*acetylation^45^. Thus, studies that use such antibodies and FACS to report on the presence of gangliosides on the cell surface such as 9-*O*-Ac-GD3 may actually also, or even mainly, be detecting 9-*O*-Ac Sia modified glycoproteins including NCL.

Although the signals for NCL and 9*-O-*Ac-Sia correlated, bovine HE^(mut)^-Fc clearly detects *O*-Ac-Sia bearing structures other than NCL on pre-B ALL cells, as visualized in the ImageStream analysis. Conversely, some cell surface NCL did not co-localize with signals from 9*-O-*Ac-Sia, supporting our biochemical analysis in which a substantial amount of glycosylated (100 kDa) NCL was found in the flow-through of CCA lectin, bovine HE^(mut)^, and porcine HE^(mut)^ affinity columns. Thus, although 9*-O-*Ac-Sia-NCL clearly constitutes a part of all the structures detected by these lectins, not all of the sialylated NCL is modified by 9*-O-*Ac and not all of the 9*-O-*Ac-Sia-bearing structures are NCL. It is therefore not surprising, that increased extracellular Ca^2+^ or lipid raft disruption by MβCD markedly affected cell surface NCL, but minimally changed 9*-O-*Ac-Sia-bearing structures.

We also investigated if the sialylation of cell surface NCL regulates its location. We found, surprisingly, that a very brief treatment with *C. perfringens* sialidase caused an increase in NCL levels detected on the plasma membrane. In principle, de-sialylation can increase the accessibility of epitopes recognized by the anti-NCL antibodies. However, on a Western blot, these antibodies detect both sialylated and non-sialylated NCL. As NCL only has a short retention time on the cell surface^33^, the detected increased level could be caused by decreased endocytosis, or accelerated exocytosis. For example, removal of terminal Sia could expose glycans on NCL that can subsequently be bound or cross-linked by lectins such as Galectin-3^46^, preventing endocytosis of NCL by its sequestration outside of lipid rafts. Increased exocytosis could also play a role, as sialylation decreases exocytosis of the lysosomal sialoglycoprotein Lamp1 (CD107a)^47^. However, it is unlikely that *C. perfringens* sialidase can enter the pre-B ALL cells to remove Sia from intracellular NCL, and therefore a stimulatory effect of this enzyme on putative NCL exocytosis via a lysosomal/endosomal route is improbable. Alternatively, lipid raft partitioning may be regulated by the sialylation state of NCL. Some *O*-glycosylated cell surface proteins including PSGL-1, CD43, and CD44 need sialylation to be able to associate with lipid rafts^48^. Because both disruption of lipid rafts using MβCD and sialidase treatment cause increased retention of NCL on the surface, an alternative explanation is, that sialidase treatment decreases NCL location in lipid rafts and by this mechanism delays endocytosis.

NCL is viewed as therapeutic target in cancer although its expression is not restricted to malignant cells. Based on gene array expression data, pre-B ALLs of different subcategories all express high levels of NCL mRNA, but not significantly higher than normal control CD19+ CD10+ pre-B cells, with the exception of the MLL subcategory (Supplementary Fig. S1). We also detected cell surface NCL protein on normal CD19+ CD10+ precursor B cells although this was lower than levels on malignant pre-B ALL ICN13 cells. Other studies have shown that NCL directly or indirectly regulates expression of a large number of genes in normal hematopoiesis: Mahotka *et al*.^49^ found significant changes in levels of around 24% of all expressed genes when they overexpressed NCL in normal human CD34+ hematopoietic stem cells. Nonetheless, as reviewed in Bates *et al*.^32^ numerous anti-NCL therapeutics including the aptamer AS1411 and its derivatives have been extensively used and even evaluated in a clinical trial^32^. AS1411 has shown surprisingly low systemic toxicity in animal models or in humans, indicating that interference with NCL activity in normal cells has few consequences or, alternatively, that the therapeutic window consists of the differential expression levels on the cell surface between normal and malignant cells. In this context it is important to note that some therapeutics such as anti-NCL antibodies^50^ are completely dependent on NCL on the plasma membrane to be able to stimulate antibody-dependent cytotoxicity of immune effector cells and that for such a therapeutic, the presence or absence of NCL on the cell surface will be a critical factor.

Our results also tentatively suggest a link between location of NCL on the plasma membrane and sensitivity to AS1411 for some of the pre-B ALLs. US7 and TXL2 were markedly less affected by AS1411 than other pre-B ALLs, and US7 and TXL2 were among the pre-B ALLs with the lowest percentage of cells with NCL cell surface expression. Conversely, LAX56 with the highest sensitivity also had a high percentage of cell surface NCL. Thus from the therapeutic anti-NCL perspective, prolongation of NCL occupancy or retention time on the cell surface would be desirable. Although we found that sialidase treatment of pre-B ALL cells would produce this effect, such a treatment cannot be applied as a therapy. In contrast, our finding that vincristine, a conventional chemotherapeutic, also produces this effect, suggests that combination treatments of vincristine with an anti-NCL agent such as AS1411 or an anti-NCL antibody may be more effective than treatment with a single agent.

## Materials and Methods

### Cell culture and cells

The murine OP9 stromal cell line (CRL-2749) and the human acute lymphoblastic leukemia cell line RS4;11 (CRL-1873) were obtained from the American Type Culture Collection. Human pre-B ALLs in this study including TXL2, US7, US7R, LAX56, LAX57, ICN3, ICN12, and ICN13 have been previously described (US7, US7R, TXL2^51–54^; LAX56, LAX57^55^, ICN3, ICN13^52^). Leukemia cells were co-cultured with irradiated, mitotically inactivated OP9 cells in MEM-α medium supplemented with 20% FBS, 1% L-glutamine and 1% penicillin/streptomycin (Life Technologies, Grand Island, NY). All human specimen collection protocols were reviewed and approved by the Children’s Hospital Los Angeles Institution Review Board (IRB) [Committee on Clinical Investigations] (CCI). Samples were collected after obtaining a written informed consent and/or as leftover specimens that were initially collected for clinical diagnostic purposes and were discarded as medical waste when no longer needed for clinical purposes. All specimens were de-identified/anonymized before acquisition for research. Collections and experiments were performed in accordance with IRB guidelines, regulations, and approvals. After red blood cell lysis, mononuclear cells were collected by Ficoll centrifugation and immediately analyzed, or viably frozen. For isolation of normal human CD19 + CD10+ pre-B precursors, viably frozen CD34-depleted CD19+ enriched normal human bone marrow samples were thawed and processed with a Release Human CD19 Positive Selection Kit and Human CD10 Positive Selection Kit (STEMCELL Technologies, Vancouver, BC, Canada).

### Preparation and assembly of lectin affinity column chromatography and proteomics analysis

*Cancer antennarius* lectin (California crab; CCA lectin) was purchased from EY Laboratories, Inc. (San Mateo, CA, USA). Porcine torovirus (PToV-P4) and bovine coronavirus (BCoV-Mebus) Hemagglutinin-Esterase (HE-Fc probes) including wild type (esterase active, HE^(wt)^ -Fc) and mutant (esterase inactive, binding activity to 9*-O-*Ac sialic acids, HE^(mut**)**^ -Fc) were generated as described^29^. For use in lectin affinity column chromatography, each lectin was biotinylated with EZ-Link^TM^ Sulfo-NHS-LC-Biotin (Thermo Fisher Scientific, Waltham, MA, USA) according to the manufacturer’s instructions. Biotinylated lectin was incubated with pre-washed Dynabeads® Streptavidin (Invitrogen, Waltham, MA, USA) for 2 hr at 4 °C to assemble the lectin-magnetic bead complex. For the CCA lectin affinity column, US7 and TXL2 lysates in Triton X-100 lysis buffer (TX buffer; 150 mM NaCl, 50 mM Tris, pH 7.4, 1% Triton X-100, 10% glycerol, 1 mM EDTA, 1x protease and phosphatase inhibitors [Roche, Basel, Switzerland]) were diluted in binding buffer containing 50 mM Tris-HCl (pH 7.2), 150 mM NaCl, and 50 mM calcium chloride, and incubated with lectin-magnetic bead complex at 4 °C overnight with rotating. After several washes with binding buffer, the mixture was reacted with elution buffer (200 mM sodium citrate in binding buffer) to elute target proteins (4 °C, 2 hr). The flow-through, wash, and eluate fractions were concentrated via centrifugation on a filter (MWCO 3000, Amicon Ultra-0.5, EMD Millipore, Billerica, MA, USA).

For proteomic analysis, the concentrated elution fraction from the CCA lectin affinity column was analyzed by SDS-PAGE and visualized by silver staining (Fig. 1a) or Coomassie staining. A 100 kDa band of interest was cut from the Coomassie-stained gel and analyzed at the University of Southern California Proteomics Core Facility.

For HE-Fc affinity columns, a total lysate of US7 pre-B ALL cells was incubated with a biotinylated HE^(mut)^-Fc probe at 4 °C overnight with rotating, followed by immobilization by magnetic beads for an additional 2 hr. After several washes, the elution fraction was analyzed by Western blotting.

### Enzyme treatment

To examine glycosylation of NCL, the elution fraction from the CCA lectin affinity column was incubated with *O-*sialoglycoprotease (OSGPase; Cedarlane, Burlington, NC), peptide N-glycosidase F (PNGase F; New England Biolabs, Ipswich, MA), or sialidase (from *Clostridium perfringens*, Sigma, St. Louis, MO) at 37 °C for 1 hr to overnight, according to the manufacturer’s instructions. The digested samples were concentrated and subjected to Western blotting.

We used 3 × 10^6^ US7 and ICN13 cells in 200 μl PBS per condition to study cell surface NCL and cell surface 9*-O-*Ac-Sia under various conditions. Treatment included: 2 μg bovine HE^(wt)^ -Fc, 100 mU sialidase, 1 and 5 mM methyl beta-cyclodextrin (MβCD), 1 mM CaCl_2_, and 5 mM EGTA at 37 °C for 1 hr. After washing twice with PBS, cells were analyzed by flow cytometry. For the time-course sialidase treatment, 1 to 2 × 10^6^ US7 pre-B ALL cells in 100 μl PBS per time point were treated with 100 mU sialidase. Control cells were incubated in 100 μl PBS without sialidase. Each set was washed with cold PBS and flow cytometry was used for cell surface NCL detection.

### Western blotting and immunoprecipitation

Cells were lysed in TX lysis buffer and incubated for 30 min on ice. Lysates were centrifuged at 14,500 rpm for 15 min at 4 °C and the supernatant was used for column chromatography, Western blotting, and immunoprecipitation. Antibodies for Western blotting include: NCL (cat. sc-8031, Santa Cruz Biotechnology, Dallas, Texas) which was used for all immunoprecipitations (IPs) and flow cytometric analysis; NCL (cat. ab22758, Abcam, Cambridge, MA) also used for the WB in Fig. 2d and Fig. 5b; *O-*acetyl GD3 monoclonal antibody D1.1 (cat. 37-9100, Invitrogen, Grand Island, NY); and anti-CDw60 antibody M-T6004 (cat. ab22384, Abcam). Also, 10 μg of porcine torovirus or bovine coronavirus HE^(mut)^ -Fc was used for immunoblotting. Western blotting of membranes with β-actin antibody (cat. sc-47778, Santa Cruz Biotechnology) was used as a loading control. For regular immunoprecipitations, 500 μg lysate was incubated with 5 μg mouse anti-NCL (cat. sc-8031, Santa Cruz Biotechnology) overnight (1 μg IgG - negative control), followed by additional incubation with precleared protein G for 1 hr with rotating. The denatured IP product was subjected to Western blotting.

To analyze 9*-O-*Ac-Sia on the cell surface and in the cytoplasm, pre-B ALL cells were biotinylated. Ten to fifty million US7 and ICN13 cells were washed in cold DPBS, biotinylated for 2 hr at 4 °C, and lysed in TX lysis buffer. Prewashed NeutrAvidin agarose (Pierce, Waltham, MA) was incubated with biotinylated cell lysate for 2 hr at 4 °C with gentle rotating. After a first centrifugation, the supernatant was collected as the intracellular fraction, and the resin was washed with TX buffer several times. For cell surface fractionation, 200 μl of 1 M glycine (pH 2.8) was added, the resin was incubated for 10 min and then the reaction neutralized by adding 20 μl of 1 M Tris (pH 8.0). Each fraction (intracellular and cell surface) was used for a standard immunodetection IP.

### Flow Cytometry and Image Stream

Cell surface NCL and 9*-O-*Ac-Sia were measured by flow cytometry. The NCL monoclonal antibody (cat. sc-8031) was labeled using a Mix-n-Stain™ CF™ 647 antibody labeling kit (MX647, Sigma, St. Louis, MO). The mutant bovine or porcine HE^(mut**)**^ -Fc probe was allowed to bind to cell surface 9*-O-*Ac-Sia first, after which FITC anti-human Fc IgG antibody (cat. 409309, Biolegend, San Diego, CA) were used to detect its binding as follows: pre-B ALL cells were first blocked using human FcR blocking reagent (cat. 130-059-901, MACS Milteny Biotec, San Diego, CA), then fixed with IC fixation buffer (cat. 00-8222-49, eBioscience, Waltham, MA) for 15 min at RT. Cells were next incubated with 1 μg of each antibody or probe per 1 × 10^6^ cells for 1 hr at 4 °C, and analyzed on an Accuri C6 cytometer (BD Biosciences, San Jose, CA). As a control, cells were stained with isotype-matched IgG conjugated with fluorophore (IgG-FITC) or incubated with secondary antibody but no lectin. This experiment was performed two-three times independently for every ALL.

To evaluate cell surface NCL and 9-*O*-Ac-Sia in viably frozen CD34-depleted, CD19-enriched normal human bone marrow samples, cells were blocked, fixed, and incubated with the following antibodies: FITC anti-human CD19 (cat. 302206, clone HIB19, Biolegend) or APC anti-human CD19 (cat. 302212, clone HIB19, Biolegend), PE anti-human CD10 (cat. 555375, clone HI10a, BD Bioscience), NCL (cat. sc-8031, clone C23/MS-3, Santa Cruz)- CF647, and porcine HE^(mut)^-Fc followed by FITC anti-human Fc IgG antibody. Cells were analyzed using an LSR FortessaTM X-20 (BD, Franklin Lakes, NJ). The percentage in each population positive for cell surface NCL and 9-*O*-Ac-Sia was calculated in the gate of double-positive populations for CD19+ and CD10+.

To image co-localization of cell surface NCL and 9*-O-*Ac-Sia, cells were blocked, fixed, and co-stained with NCL-CF647 and HE^(mut**)**^-Fc -CF488, followed by analysis on an Image Stream (ImageStream®X Mark II Imaging Flow Cytometer, Amnis, EMD Millipore, Billerica, MA). Image files of 5,000 to 20,000 events were acquired. IDEAS 6.2 software was used to analyze the co-localization of NCL and 9-*O*-Ac-Sia on the plasma membrane of cells that were positive for both.

### Drug treatment

US7 cells were co-cultured with irradiated OP9 in 6-well plates in the presence of 5 nM vincristine for the indicated time period. Fresh media with drug was supplied every 2–3 days. Total cell number and cell viability were determined using Trypan blue exclusion. FACS was used to evaluate cell surface levels of NCL and 9*-O-*Ac-Sia during drug treatment. Treatments were carried out on triplicate samples in three independent experiments. For NCL-specific treatment, AS1411 (5′-GGTGGTGGTGGTTGTGGTGGTGGTGG-3′) and control CRO26 (5′-CCTCCTCCTCCTTCTCCTCCTCCTCC-3′) aptamers were obtained from Invitrogen and from IDT (Coralville, Iowa). Cells were incubated with various concentrations as indicated, for 48 or 72 hr, and Trypan blue exclusion was used to count live cells.

### Statistical analysis

Statistical analysis was performed with Prism 5.0 or 7.0 software. Data are presented as mean ± S.E. Statistical significance of differences between groups was evaluated using student t-test, one-way or two-way ANOVA as indicated. A value of *p* < 0.05 is considered to be statistically significant.

## Electronic supplementary material

Supplementary Figures

## Data Availability

All data generated or analyzed during this study are included in this published article and its Supplementary Information files.
