# Supplementary material for: Pre-B acute lymphoblastic leukemia expresses cell surface nucleolin as a 9-O-acetylated sialoglycoprotein
Source: Sci Rep. 2018 Nov 21;8:17174. doi: 10.1038/s41598-018-33873-2 (PMC6249323; doi:10.1038/s41598-018-33873-2)
Supplement: Supplementary file 1 — Supplementary Figures [file 41598_2018_33873_MOESM1_ESM.pdf]

Pre-B acute lymphoblastic leukemia expresses cell surface nucleolin as a 9-*O*-acetylated sialoglycoprotein

SUPPLEMENTARY DATA

Eun Ji Joo[1], Brian R Wasik[2], Colin Parrish[2], [&]Helicia Paz[3], Martina Mühlenhoff[4], Hisham Abdel-Azim[5], John Groffen [3,6] and [*]Nora Heisterkamp[1, 5, 6]

1 Department of Systems Biology, Beckman Research Institute, City of Hope, Monrovia, CA, USA
2 Department of Microbiology and Immunology, Baker Institute for Animal Health and Feline Health Center, Cornell University, Ithaca, NY, USA
3 Section of Molecular Carcinogenesis, The Saban Research Institute of Children's Hospital Los Angeles, Los Angeles, CA, USA
4 Institute of Clinical Biochemistry, Hannover Medical School, Hannover, Germany
5 Division of Hematology/Oncology and Bone Marrow Transplant, Children's Hospital Los Angeles, Los Angeles, CA, USA
6 Departments of Pediatrics and Pathology, Keck School of Medicine, University of Southern California, Los Angeles, CA, USA

* Correspondence to: Nora Heisterkamp, email: eheisterkamp@coh.org Department of Systems Biology, Beckman Research Institute, City of Hope, 1218 South Fifth Avenue, Monrovia, CA 91016. Tel: 626-218-7503

[&] Current address University of California, Los Angeles, Los Angeles, CA 90095

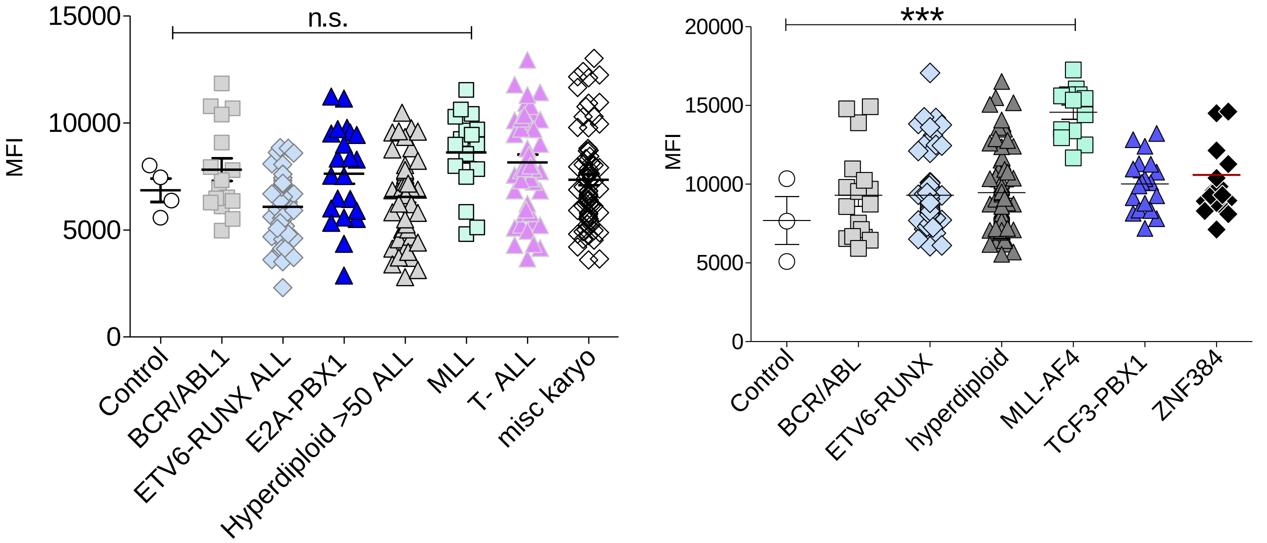

Figure S1. *NCL* mRNA expression. Meta-analysis of expression of human acute lymphoblastic leukemias by genetic lesions compared to control normal bone marrow CD19+CD10+ precursor cells. MFI, mean fluorescent intensity. Each symbol represents the value of one patient sample. Left panel, 270 patient samples and CD19+CD10+ normal bone marrow controls, Coustan-Smith et al (GSE28497); right panel, 229 patient samples Hirabayashi et al. (GSE79533). Graphs reports mean ± S.E. ***$p<0.001$. n.s., not significant. One-way Anova.

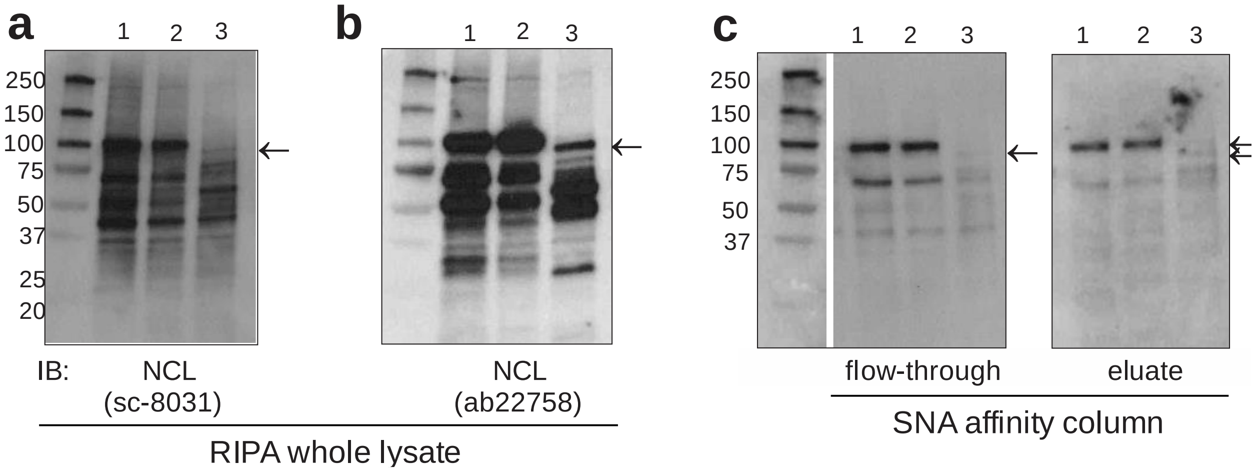

Figure S2. The 100 kDa NCL protein is sialylated. US7 pre-B ALL RIPA buffer lysates (**a**, **b**) or the flow-through or eluate of a SNA lectin affinity column (**c,** immunoblotted with sc-8031) were left as is (lanes 1) or incubated in buffer (PBS) without (lanes 2) or with (lanes 3) 100 mU of *C. perfringens* sialidase for 90 min. An arrow points to the fully sialylated/glycosylated 100 kDa NCL protein.

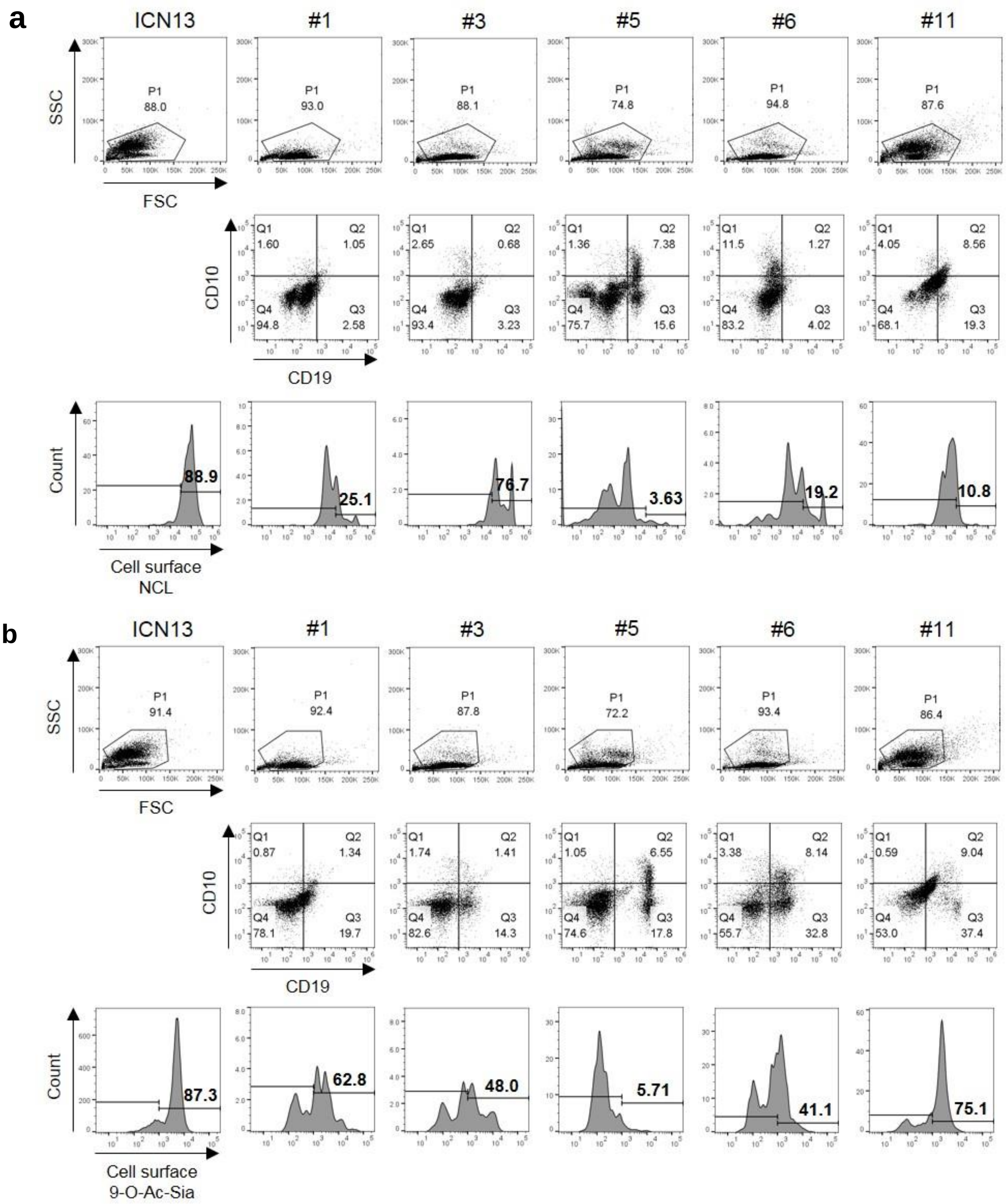

Figure S3. Cell surface NCL and cell surface 9-*O*-Ac-Sia in normal BM samples. Five viably frozen CD34-depleted CD19+-enriched normal human bone marrow (BM) samples were prepared as described in *Materials and Methods* and compared to pre-B ALL ICN13 for cell surface NCL (**a**) and cell surface 9-*O*-Ac-Sia (**b**). Histograms were generated from the gated population of cells double positive for CD19 and CD10 (Q2 in quadrant graph).

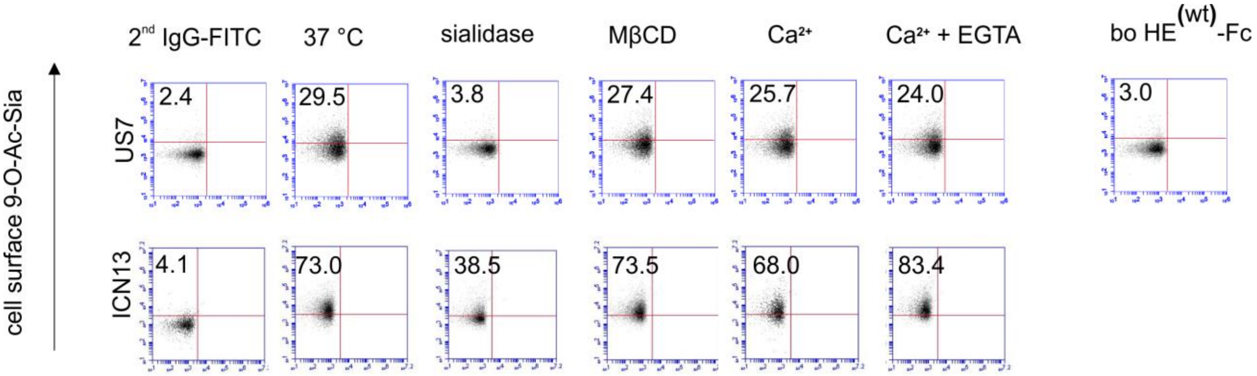

Figure S4. Regulation of 9-*O*-Ac-Sia structures on the surface of pre-B ALL cells. US7 and ICN13 cells as indicated were treated with nothing (control, 37°C), sialidase, MβCD, exposed to extracellular Ca^{2+} or Ca^{2+} and EGTA as described in *Materials and Methods* and then assayed for cell surface 9-*O*-Ac-Sia. Bo $HE^{(wt)}$-Fc treatment, control. Numbers in the upper left quadrant indicate the percentage positive cells.

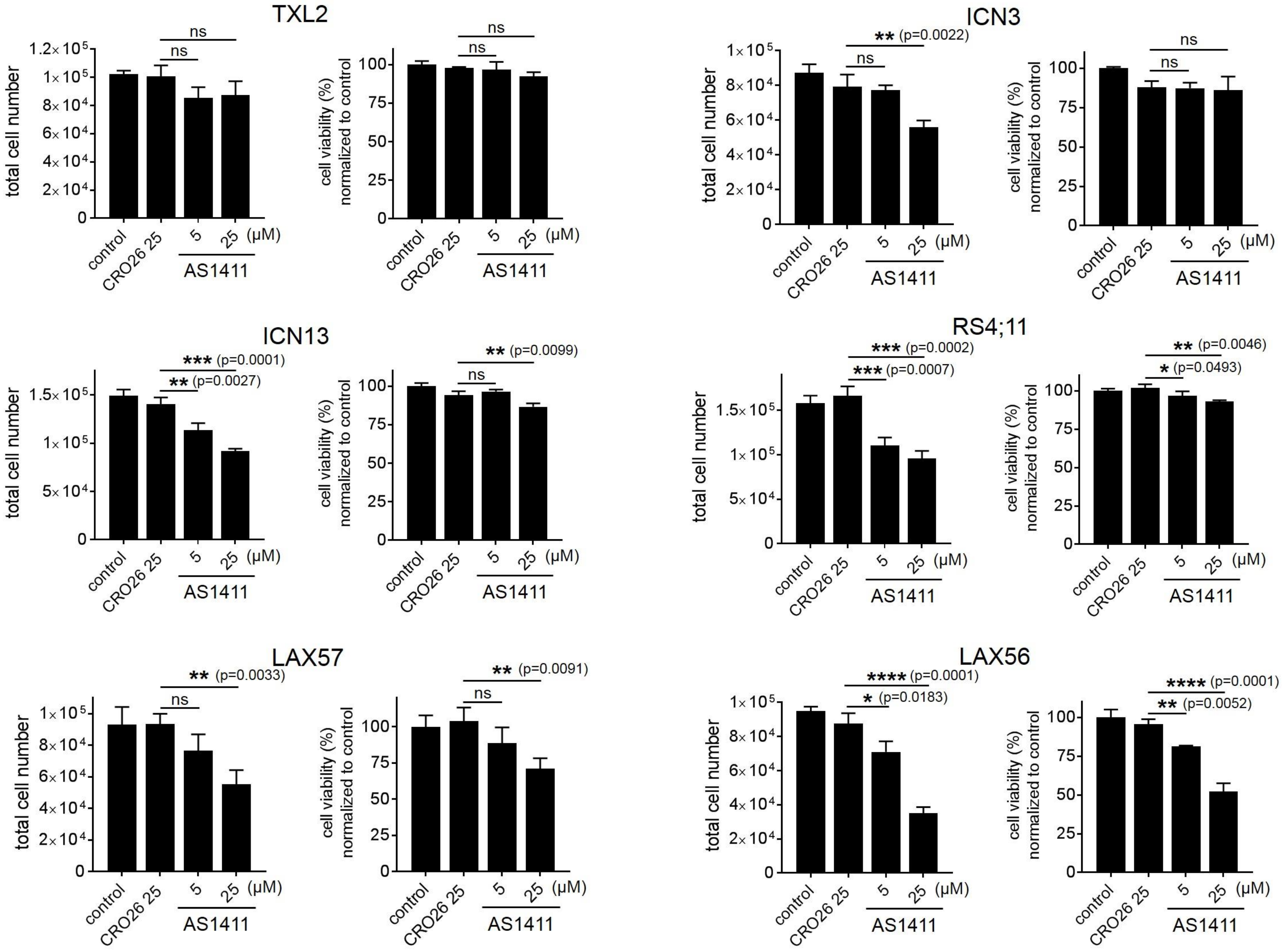

Figure S5. Treatment of pre-B ALLs with anti-NCL aptamer AS1411. Pre-B ALLs TXL2, ICN3, ICN13, RS4;11, LAX57, and LAX56 as indicated were compared to each other for sensitivity to treatment with 5 or 25 μM AS1411 or 25 μM CRO26 control aptamers for 48 hr in the absence of stroma. Total cell numbers (left panels) or viability (Trypan blue excluding cells/total cell number x 100)(right panels) determined for all samples on triplicate wells. Values: mean ± S.E. ns, not significant, *p<0.05, **p<0.01, ***p<0.001. One-way ANOVA.

Figure 1b

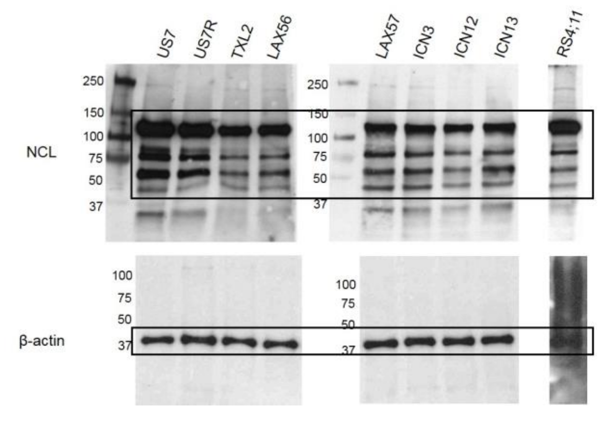

Figure 2a

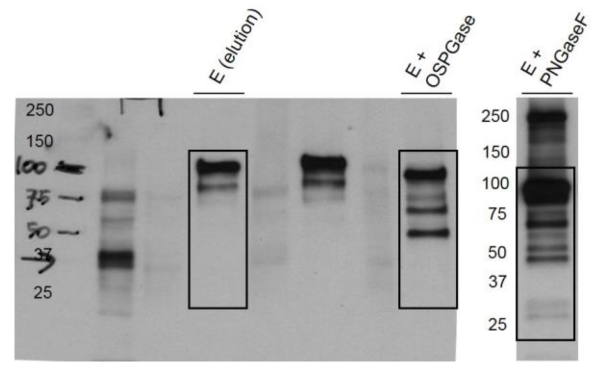

Figure 2b

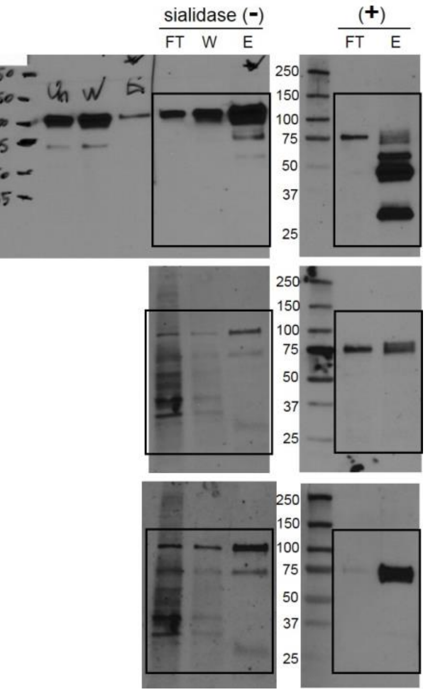

Figure 2c

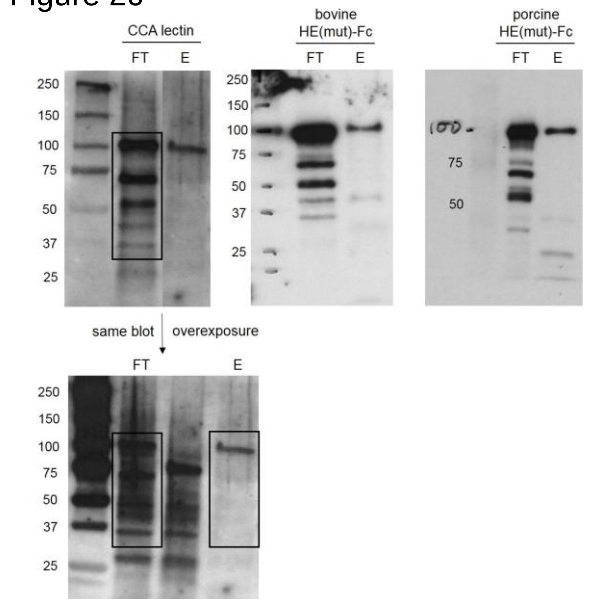

Figure 2d

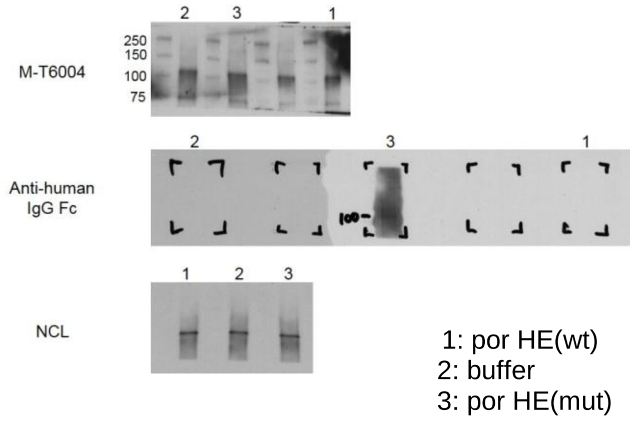

Figure 3a

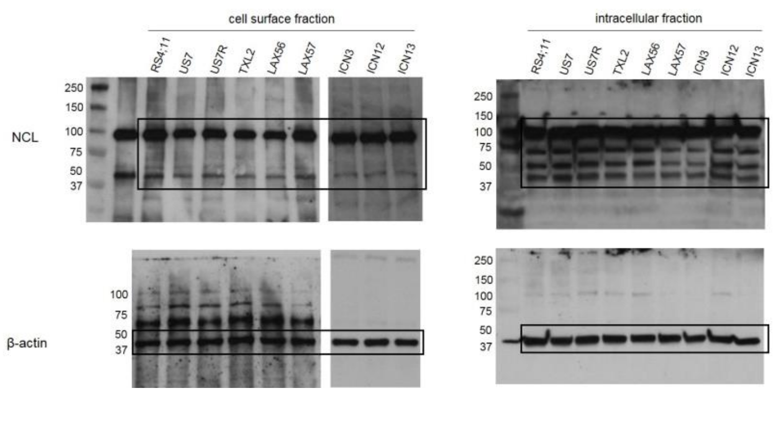

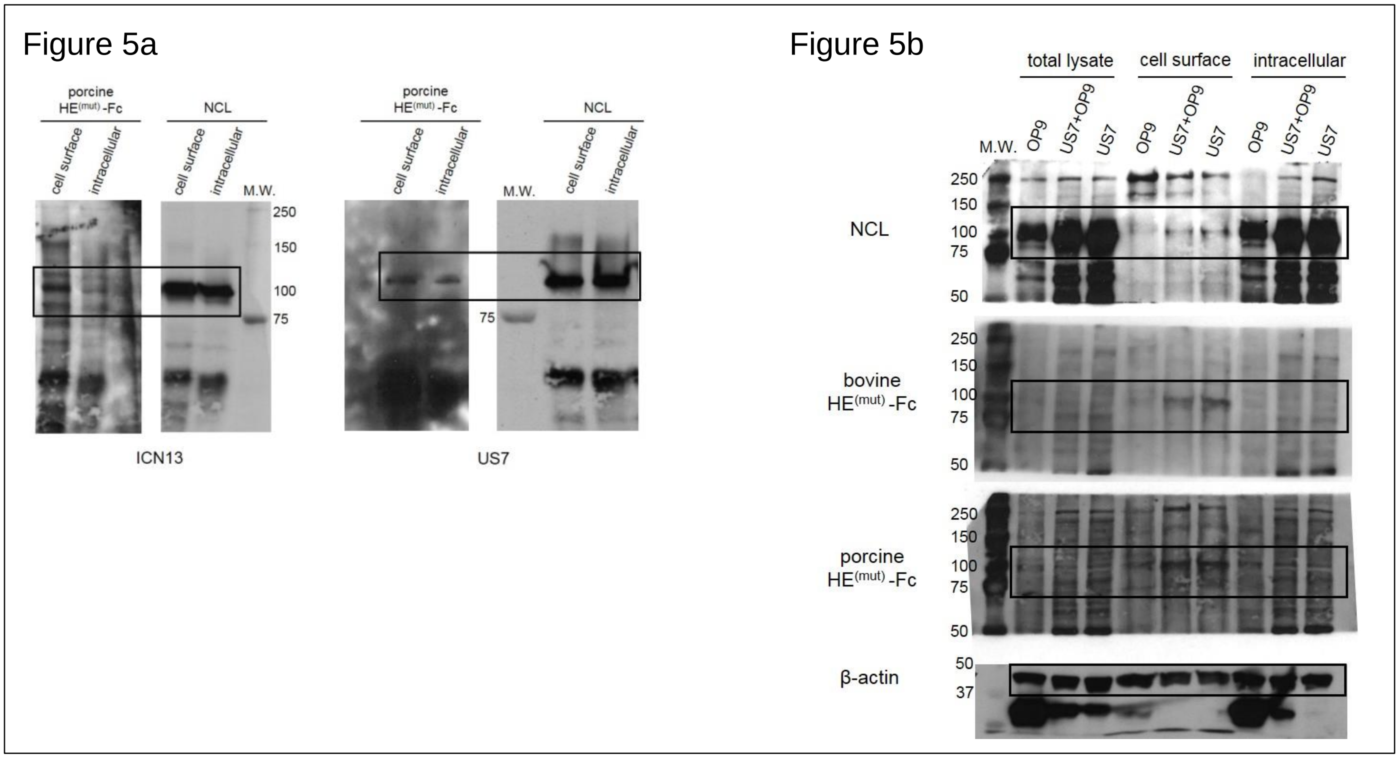

Figure S6. Original images of immunoblots in main figures. Uncropped images are presented with molecular weight (M.W.) marker. Each membrane was incubated with the indicated antibodies to detect the target molecules showing in the boxed regions.
